# Velocity Loss Thresholds Reliably Control Kinetic and Kinematic Outputs during Free Weight Resistance Training

**DOI:** 10.3390/ijerph17186509

**Published:** 2020-09-07

**Authors:** Madison Pearson, Amador García-Ramos, Matthew Morrison, Carlos Ramirez-Lopez, Nicholas Dalton-Barron, Jonathon Weakley

**Affiliations:** 1 School of Behavioural and Health Sciences, Australian Catholic University, Brisbane 4014, Queensland, Australia; matthew.morrison@myacu.edu.au (M.M.); jonathon.weakley@acu.edu.au (J.W.); 2 Department of Sports Sciences and Physical Conditioning, Faculty of Education, Universidad Católica de la Santísima Concepción, Concepción 2850, Chile; amagr@ugr.es; 3 Department of Physical Education and Sport, Faculty of Sport Sciences, University of Granada, 18010 Granada, Spain; 4 Institute for Sport, Physical Activity and Leisure, Leeds Beckett University, Leeds LS6 3GZ, West Yorkshire, UK; c.ramirez@leedsbeckett.ac.uk (C.R.-L.); N.E.Dalton-Barron@leedsbeckett.ac.uk (N.D.-B.); 5 Yorkshire Carnegie Rugby Union Club, Kirkstall Training Ground, Leeds Rugby Academy, Leeds LS6 3BR, West Yorkshire, UK; 6 England Performance Unit, The Rugby Football League, Leeds LS6 3GZ, West Yorkshire, UK

**Keywords:** velocity-based training, reliability, resistance training, training prescription

## Abstract

Exercise velocity and relative velocity loss thresholds (VLTs) are commonly used in velocity-based resistance training. This study aims to quantify the between-day reliability of 10%, 20%, and 30% VLTs on kinetic and kinematic outputs, changes in external load, and repetition characteristics in well-trained athletes. Using a repeated, counter-balanced crossover design, twelve semi-professional athletes completed five sets of the back squat with an external load corresponding to a mean concentric velocity of ~0.70 m·s^−1^ and a VLT applied. The testing sessions were repeated after four weeks of unstructured training to assess the long-term reliability of each VLT. A coefficient of variation (CV) <10% was used to classify outputs as reliable. Kinetic and kinematic outputs and external load were largely reliable, with only peak power during sets 2–5 within the 10% VLT condition demonstrating a CV >10% (CV: 11.14–14.92%). Alternatively, the repetitions completed within each set showed large variation (CV: 18.92–67.49%). These findings demonstrate that by utilizing VLTs, kinetic and kinematic outputs can be prescribed and replicated across training mesocycles. Thus, for practitioners wishing to reliably control the kinetic and kinematic stimulus that is being applied to their athletes, it is advised that a velocity-based approach is used.

## 1. Introduction

Velocity based training is a method of resistance training that supports the appropriate prescription of exercise intensity and volume [1,2,3,4]. The mean concentric velocity of the first repetition of a set can be used to prescribe the external load (i.e., training intensity), due to the near perfect inverse relationship between movement velocity and the relative load (i.e., % of the 1-repetition maximum (1RM) [4,5,6,7,8,9]. Additionally, velocity loss thresholds (VLTs) can be implemented to support the prescription of training volumes, with differing VLTs inducing varying physical and physiological adaptations [2]. For example, Pareja-Blanco et al. demonstrated that a 20% VLT promotes greater strength and power adaptations than a 40% VLT [2]. However, greater hypertrophic responses occur with a 40% VLT. These divergent responses have been attributed to alterations in the kinetic and kinematic (i.e., force, power, and velocity) outputs that are produced when using this prescriptive method [2,10,11]. 

Velocity loss thresholds have been shown to mitigate the differences in physiological characteristics (e.g., strength endurance) during resistance training [11]. It has recently been shown that by applying VLTs, kinetic and kinematic outputs can be closely regulated across multiple sets of the back squat within a single training session [11]. However, it should be acknowledged that the replication of this control of outputs across training sessions has not been investigated. In addition, due to the fluctuating nature of fitness and fatigue, it is unknown how the external load and number of repetitions change across sessions when VLTs are implemented [1,11]. Thus, for practitioners to be able to confidently apply VLTs to help mitigate differences in athlete heterogeneity during resistance training, the between-session reliability of these thresholds on kinetic, kinematic, external load, and repetition characteristics needs to be established. 

Recent studies have shown that velocity-based training and the use of VLTs promote superior physical adaptations when compared to percentage-based training [12,13]. Furthermore, velocity-based training has been suggested to allow practitioners to have greater control over the kinetic and kinematic outputs over multiple sets [10,11,14]. However, for practitioners to be able to confidently implement VLTs across training mesocycles, the between-day reliability must be known. Therefore, this study aimed to investigate the between-day reliability of force, power, and velocity outputs from well-trained athletes across five sets of the free-weight back squat when 10%, 20%, and 30% relative VLTs are applied. Furthermore, changes in the number of repetitions and external loads were assessed. 

## 2. Materials and Methods

### 2.1. Experimental Design 

This study utilized a repeated, counterbalanced, crossover design to assess the long-term between-day reliability of different VLTs on: (I) mean and peak kinetic and kinematic outputs and (II) changes in the number of repetitions and external load characteristics. Following a familiarization session, participants were randomly assigned to six testing sessions [15]. For the first three testing sessions, participants performed five sets of the back squat, with each session utilizing a different VLT (i.e., 10%, 20%, and 30%). For all VLTs, the first repetition of the initial set had a mean concentric velocity of 0.70 ± 0.01 m·s^−1^. For the remaining sets, the initial repetition velocity was 0.70 ± 0.06 m·s^−1^. Each set was terminated according to the VLT applied. Between sets, three minutes of passive recovery was provided. At least 72 h recovery was provided between each of the first three testing sessions. The remaining three testing sessions were completed after four weeks of unstructured training. Each participant completed a 10%, 20%, and 30% VLT for a second time in the same order. The four-week period that separated each VLT of the same type allowed for the long-term reliability of each VLT to be assessed. All six sessions were conducted at the same time of day, and participants were required to avoid any strenuous exercise 48 h prior to each session. 

### 2.2. Participants 

Twelve male semi-professional rugby union athletes (mean ± standard deviation; age: 23.1 ± 2.4 years; body mass: 88.8 ± 13.3 kg; height: 180 ± 7 cm) from a British University and Colleges Super Rugby club (United Kingdom) volunteered to participate in this study. All participants were required to have at least two years of resistance training experience with the back squat exercise [16]. Additionally, during the six months preceding the study, participants had been training the back squat at least twice a week, with intensities between 60–93% of 1RM [11]. All testing was done during the rugby union off-season, to prevent interference with training schedules. During the familiarization session, the study protocols were explained, all questions from the participants were answered, and written informed consent was collected. The back squat exercise was also demonstrated during this session to assess and standardize the technique used across all testing protocols. 

The Leeds Beckett University Ethics Committee approved all protocols in accordance with the Declaration of Helsinki (number 47590).

### 2.3. Procedures

Each testing session commenced with a standardized dynamic warm up, followed by a squat-specific warm up [17]. This specific warm up consisted of eight repetitions with an empty 20 kg barbell (Eleiko Sport AB, Halmstad, Sweden), followed by three sets of 3–5 repetitions at self-selected submaximal loads, based on the athlete’s choice [18]. All repetitions were required to meet strict squat technique; whereby the top of the knee became parallel with the fold between the torso and thigh (assessed by the primary investigator) [16]. The mean concentric velocity of all warm-up repetitions was monitored by a linear position transducer (GymAware, Kinetic Performance Technology, Canberra, Australia), which has shown to be a valid and reliable device [19], and used in previous acute and longitudinal resistance training research with a similar population [19,20,21]. Visual (i.e., the mean concentric barbell velocity) and verbal (e.g., words of encouragement) feedback was also provided following every repetition to support maximal concentric intent during resistance training [22,23,24,25]. 

After the completion of the squat-specific warm up, a load that elicited a barbell mean concentric velocity of 0.70 ± 0.01 m·s^−1^ was found. To achieve this, the primary investigator (who was present for all testing occasions) placed 70% of the participant’s estimated 1RM on the barbell. The participants then performed two consecutive repetitions with this load. If the mean concentric velocity of the fastest repetition was not within 0.70 ± 0.01 m·s^−1^, three minutes of rest was provided and the external load was adjusted, according to methods previously outlined by Weakley et al. [11]. Once the load with a mean concentric velocity of 0.70 ± 0.01 m·s^−1^ was established, five minutes of rest was provided before five sets of either a 10%, 20%, or 30% velocity loss protocol was conducted. Thus, a set in a 10%, 20%, or 30% protocol would be terminated when velocity reached 0.63 m·s^−1^, 0.56 m·s^−1^, or 0.49 m·s^−1^, respectively. This was consistent across all sets and sessions. For sets 2–5, the initial repetition was required to be 0.70 ± 0.06 m·s^−1^ [11,26]. If the velocity of the first repetition from these subsequent sets was not within this range, an additional 30 s of passive rest was provided. After this rest, a single repetition was again performed. If the barbell velocity was within 0.70 ± 0.06 m·s^−1^, the set continued until the required velocity loss was achieved. If the velocity on the second attempt was still outside this ± 0.06 m·s^−1^ range, an additional 30 s of rest was supplied, while the load was adjusted by approximately 5% of the participant’s estimated 1RM [11,26]. Once adjustments were made, all participants on the second attempt were found to be able to attain a barbell velocity of 0.70 ± 0.06 m·s^−1^. 

### 2.4. Outcome Measures 

A linear position transducer was used to collect all concentric mean and peak kinetic and kinematic data with a sampling rate of 50 Hz [11]. For all sessions, the retractable cord was positioned approximately 65 cm left of the center of the barbell [27]. Information from the linear position transducer was transmitted to a tablet (iPad, Apple Inc., California, USA) via Bluetooth. This enabled data to be collected for each participant across each session. The mean and peak concentric values of force, power, and velocity were averaged for each of the five sets during the 10%, 20%, and 30% velocity loss conditions to be used in further analysis. Additionally, the external load and number of repetitions were recorded for each set.

### 2.5. Statistical Analysis

Raw kinetic, kinematic, external load, and repetition data were log transformed to allow the typical error (TE) to be calculated as a coefficient of variation (CV), using a premade Microsoft Excel spreadsheet [28]. The TE was calculated as follows:

(1) $$\mathrm{TE}\;=\;S_{\mathrm{dif}}\sqrt{2}$$

where S_dif_ is the standard deviation of the difference score between two trials [29]. Similar to previous resistance training studies, a CV of 10% or less was used to categorize a variable as reliable [30,31]. Due to the closely regulated mean concentric velocities within this study, and knowledge that intraclass correlation coefficient (ICC) values are artificially reduced when samples have greater homogeneity [29], the CV was used to guide decisions of acceptable (<10%) reliability values [32]. 

## 3. Results 

Kinetic, kinematic, external load, and repetition characteristics from the 10%, 20%, and 30% relative VLTs conditions are presented in Table 1, Table 2, and Table 3, respectively.

## 4. Discussion 

This study investigated the between-day reliability of force, power, and velocity outputs across five sets of the free-weight back squat in well-trained athletes when 10%, 20%, and 30% relative VLTs were applied. Furthermore, changes in repetition number and external loads were assessed. Findings from this study show that concentric kinetic and kinematic outputs in all protocols were largely reliable (CV: <10%); with only peak power during sets 2–5 within the 10% VLT condition above this threshold (CV: 11.14–14.92%). Additionally, despite the time between testing occasions, external load demonstrated little variance. However, the number of repetitions were highly variable within conditions (CV: 18.92–67.49%). These findings demonstrate that VLTs allow for reliable prescription of kinetic and kinematic outputs within well-trained athletes. Furthermore, by prescribing load from velocity output and accompanying these with VLTs, practitioners can confidently prescribe an external stimulus (i.e., kinetic and kinematic outputs) that can be closely replicated irrespective of recent training history. To the authors’ knowledge, this is the first study to demonstrate ecologically valid, long-term reliability of these outputs from resistance training prescription. Additionally, it challenges traditional resistance training prescription methods (i.e., prescribed repetition and set schemes) which are yet to demonstrate this reliability irrespective of recent training history.

This study demonstrates that VLTs allow the reliable prescription of kinetic and kinematic outputs across days. This is despite a four-week period of unstructured training occurring between testing occasions. This suggests that by utilizing VLTs, practitioners can prescribe each individual athlete a similar training stimulus irrespective of recent training history. This is made possible by emphasizing the external stimulus (i.e., force, power, and velocity), rather than set and repetition schemes that do not account for athlete differences [33,34,35] and have been shown to cause divergent adaptations despite homogenous groups of athletes [36]. This supports previous work from Weakley et al. that demonstrated that VLTs control for differences (e.g., strength and muscular endurance) between participants and allow for homogenous training outputs within training sessions [11]. Thus, when paired with the current findings, it is now evident that various VLTs can be used to control the kinetic and kinematic outputs within and between athletes, and within and between training sessions. These findings should be of particular interest for practitioners who are wanting to closely control the external stimulus that is being applied across resistance training mesocycles.

While external load was relatively consistent across sessions (CV range: 2.38–9.22%); there were extremely large amounts of variance in the number of repetitions completed within each protocol (CV range: 18.92–67.49%). This supports the notion that prescribed repetition ranges can induce differing rates of neuromuscular fatigue, despite the same session being prescribed, and that relative VLTs can enable greater prescriptive control [11,30]. This is likely due to an attenuation of the effects of fitness and fatigue, influence of recent training history, and extraneous variables (e.g., sleep and diet) [1,37,38]. Thus, the monitoring of velocity loss during resistance training programs can ensure consistent training outcomes, and account for daily changes in physical and extraneous variables. 

While this study provides further evidence for velocity-based training and the implementation of VLTs, there are limitations. First, it is acknowledged that different starting velocities (e.g., 0.40 m·s^−1^ vs. 0.70 m·s^−1^) can affect the external load, kinetic and kinematic outputs, and the number of repetitions completed [39,40]. Despite this, VLTs are likely to demonstrate similar levels of reliability irrespective of starting velocity. Previous research by Pareja-Blanco et al. has demonstrated the effects of different starting velocities on kinematic outputs, and shown similar trends as training progresses across a mesocycle [40]. Furthermore, due to the closely regulated velocities that support the initiation and termination of a set, it is unlikely that the starting velocity will drastically alter reliability values. 

Second, while VLTs have been shown to control external kinetic and kinematic outputs, the short-term fatigue responses (e.g., neuromuscular) to this form of resistance training prescription are yet to be established. Therefore, future research should investigate whether these homogenous kinetic and kinematic outputs can induce consistent changes in performance outcomes following training (e.g., 24 h post-training). Finally, it should be acknowledged that the participants’ training experience may have improved the reliability of the external load and subsequent kinetic outputs. However, it should be noted that this would not influence velocity outputs, due to the closely regulated thresholds. 

## 5. Conclusions

In conclusion, VLTs allow for reliable prescription of kinetic and kinematic outputs during resistance training in well-trained athletes. This is despite recent training history and varying levels of muscular strength. However, it was found that a large amount of variance occurred in the number of repetitions per set. This suggests that, despite similar relative external loads between testing occasions, the rates of velocity loss and neuromuscular endurance can greatly differ. Therefore, practitioners should consider using velocity to prescribe the external load, accompanied with VLTs to control the absolute volume of training that is completed. These findings challenge the notion of predefined resistance training set and repetition schemes (e.g., 3 × 6), that are based off a given percentage (e.g., 85% of 1RM), and lend further support to a velocity-based approach.

### Practical Application 

It is recommended that VLTs are used during the prescription of resistance training for well-trained athletes. This is due to the highly reproducible kinetic and kinematic outputs that are demonstrated across prolonged periods of time. This allows for the accurate prescription of these variables and the ability to prescribe a given load (relative to 1RM). For example, a practitioner can prescribe two resistance training sessions with a 20% VLT and expect to see a mean concentric power output that demonstrates less than 5% variance, despite recent training history. However, the number of repetitions that are completed will likely vary. This may be attributed to changes in fitness and fatigue, or extraneous variables (e.g., sleep and diet). Thus, practitioners who wish to reliably prescribe the kinetic and kinematic stimulus that is applied to each athlete when training are advised to utilize velocity for the prescription of training intensities and volumes.

## Figures and Tables

**Table 1:** Kinetic, kinematic, external load, and repetition characteristics from the 10% velocity loss threshold (VLT) condition.

	External Load (kg)	Repetitions	Concentric Mean Velocity (m·s^−1^)	Concentric Peak Velocity (m·s^−1^)	Concentric Mean Force (N)	Concentric Mean Power (W)	Concentric Peak Force (N)	Concentric Peak Power (W)
**Set 1**								
Session 1	110.15±22.62	5.09 ± 3.43	0.66 ± 0.02	1.27 ± 0.10	1970.68 ± 339.99	1279.78 ± 231.00	2816.02 ± 493.69	3084.93 ± 541.68
Session 2	112.37 ± 25.70	4.83 ± 2.68	0.66 ± 0.03	1.33 ± 0.11	2009.18 ± 372.98	1319.00 ± 250.64	2914.06 ± 539.65	3326.63 ± 792.50
TE (CV% (90%CI))	3.56 (3.20 (2.38–5.00))	2.22 (44.73 (31.84–77.40))	0.02 (3.13 (2.33–4.89))	0.07 (5.18 (3.85–8.15))	34.77 (1.75 (1.30–2.72))	38.75(2.98 (2.22–4.66))	86.12 (3.01 (2.24–4.70))	298.23 (9.31 (6.88–14.80))
**Set 2**								
Session 1	109.51 ± 22.66	4.23 ± 2.53	0.65 ± 0.02	1.26 ± 0.10	1970.50 ± 343.87	1273.51 ± 231.53	2796.98 ± 529.59	2985.15 ± 615.62
Session 2	112.37 ± 25.70	4.02 ± 2.80	0.66 ± 0.02	1.31 ± 0.13	2000.21 ± 369.48	1310.67 ± 243.77	2874.70 ± 576.27	3225.88 ± 862.51
TE (CV% (90%CI))	6.41 (5.78 (4.29–9.11))	1.39 (33.60 (24.18–56.70))	0.02 (2.87 (2.14–4.48))	0.08 (6.09 (4.52–9.60))	68.56 (3.45 (2.57–5.41))	23.24 (1.80 (1.34–2.80))	151.8 (5.35 (3.98–8.42))	361.04 (11.63 (8.58–18.61))
**Set 3**								
Session 1	109.16 ± 22.12	4.61 ± 2.12	0.66 ± 0.02	1.28 ± 0.08	1962.85 ± 338.25	1285.85 ± 240.78	2776.11 ± 520.72	3087.21 ± 643.54
Session 2	113.83 ± 24.46	3.66 ± 2.29	0.65 ± 0.04	1.30 ± 0.16	2017.29 ± 347.42	1299.24 ± 217.53	2890.94 ± 497.58	3250.34 ± 811.18
TE (CV% (90%CI))	6.54 (5.87 (4.35–9.24))	1.95 (47.36 (33.63–82.43))	0.03 (3.82 (2.84–5.98))	0.11(8.62 (6.38–13.68))	69.08 (3.47 (2.58–5.43))	42.17 (3.26 (2.43–5.10))	164.32 (5.80 (4.31–9.14))	472.55 (14.92 (10.96–24.06))
**Set 4**								
Session 1	108.61 ± 23.07	4.17 ± 2.55	0.66 ± 0.03	1.29 ± 0.07	1962.65 ± 351.65	1279.54 ± 244.19	2785.5 ± 516.39	3121.77 ± 623.95
Session 2	112.99 ± 24.29	3.97 ± 2.83	0.65 ± 0.02	1.30 ± 0.14	2006.75 ± 355.15	1301.33 ± 242.90	2888.89 ± 523.71	3234.20 ± 888.35
TE (CV% (90%CI))	5.16 (4.66 (3.46–7.31))	2.53(62.04 (43.46–111.37))	0.03 (3.89 (2.89–6.09))	0.09 (6.71 (4.97–10.59))	50.34 (2.54 (1.89–3.96))	37.06 (2.87 (2.14–4.49))	136.40 (4.81 (3.57–7.55))	354.11 (11.14 (8.22–17.80))
**Set 5**								
Session 1	107.99 ± 22.93	4.42 ± 2.86	0.65 ± 0.02	1.27 ± 0.08	1955.74 ± 344.56	1261.23 ± 225.28	2752.70 ± 516.49	3058.74 ± 698.93
Session 2	113.65 ± 24.13	3.58 ± 2.47	0.65 ± 0.03	1.31 ± 0.14	2011.16 ± 356.64	1301.70 ± 231.54	2879.54 ± 536.25	3235.50 ± 892.22
TE (CV% (90%CI))	3.90 (3.52 (2.62–5.52))	2.69 (67.49 (47.05–122.49))	0.02 (3.67 (2.73–5.74))	0.09 (6.83 (5.06–10.78))	37.98 (1.91 (1.43–2.99))	36.58 (2.85 (2.13–4.46))	117.96 (4.19 (3.12–6.57))	363.60 (11.56 (8.52–18.48))

Mean ±: Standard Deviation. TE: Typical Error: CV%: Coefficient of Variation. 90% CI: 90% Confidence Interval.

**Table 2:** Kinetic, kinematic, external load, and repetition characteristics from the 20% VLT condition.

	External Load (kg)	Repetitions	Concentric Mean Velocity (m·s^−1^)	Concentric Peak Velocity (m·s^−1^)	Concentric Mean Force (N)	Concentric Mean Power (W)	Concentric Peak Force (N)	Concentric Peak Power (W)
**Set 1**								
Session 1	109.44 ± 22.23	8.25 ± 3.46	0.64 ± 0.03	1.16 ± 0.25	1963.93 ± 340.13	1230.29 ± 211.74	2830.60 ± 507.08	2894.45 ± 461.14
Session 2	106.00 ± 24.32	8.82 ± 4.73	0.64 ± 0.02	1.19 ± 0.30	1932.79 ± 369.63	1216.79 ± 223.35	2742.6 ± 496.30	2992.12 ± 583.68
TE (CV% (90%CI))	9.93 (9.22 (6.81–14.65))	2.91 (34.17 (24.58–57.74))	0.02 (2.54 (1.89–3.97))	0.06 (4.82 (3.58–7.58))	97.49 (5.00 (3.72–7.87))	42.58 (3.48 (2.59–5.45))	161.77 (5.81 (4.31–9.15))	224.73 (7.64 (5.66–12.09))
**Set 2**								
Session 1	110.35 ± 21.82	7.52 ± 2.71	0.63 ± 0.03	1.22 ± 0.10	1970.95 ± 330.79	1229.44 ± 205.34	2844.94 ± 486.05	2920.95 ± 518.23
Session 2	109.09 ± 23.72	7.09 ± 3.37	0.63 ± 0.01	1.24 ± 0.15	1964.62 ± 361.25	1233.97 ± 229.44	2798.92 ± 506.57	2967.60 ± 650.52
TE (CV% (90%CI))	4.18 (3.81 (2.83–5.96))	2.52 (34.53 (24.83–58.40))	0.03 (4.36 (3.5–6.85))	0.05 (4.26 (3.17–6.68))	44.64 (2.27 (1.69–3.54))	49.55(4.02 (2.99–6.31))	110.08 (3.90 (2.90–6.11))	199.81(6.79 (5.03–10.72))
**Set 3**								
Session 1	110.25 ± 22.59	6.56 ± 2.27	0.62 ± 0.02	1.23 ± 0.12	1969.65 ± 338.67	1200.82 ± 193.28	2802.47 ± 70.83	2971.42 ± 529.17
Session 2	110.07 ± 22.79	7.04 ± 2.45	0.62 ± 0.02	1.23 ± 0.14	1971.72 ± 352.24	1219.06 ± 206.91	2790.89 ± 477.36	2946.46 ± 645.23
TE (CV% (90%CI))	3.39 (3.07 (2.29–4.81))	2.19 (32.29 (23.27–54.32))	0.02 (3.99 (2.97–6.25))	0.07 (5.72 (4.25–9.02))	40.71(2.07 (1.54–3.22))	51.37(4.25 (3.16–6.66))	123.91(4.43 (3.29–6.95))	270.86 (9.15 (6.77–14.55))
**Set 4**								
Session 1	111.07 ± 22.85	5.14 ± 3.20	0.60 ± 0.02	1.22 ± 0.12	1980.57 ± 344.89	1181.76 ± 221.71	2824.91 ± 474.26	2934.69 ± 626.67
Session 2	110.07 ± 22.79	5.70 ± 2.71	0.62 ± 0.01	1.24 ± 0.14	1972.04 ± 351.77	1205.15 ± 210.12	2770.58 ± 453.19	2988.80 ± 667.23
TE (CV% (90%CI))	3.32(3.00 (2.23–4.69))	1.32 (24.38 (17.72–40.25))	0.02 (2.54 (1.90–3.97))	0.05 (4.27 (3.18–6.70))	43.45(2.20 (1.64–3.43))	37.78 (3.17 (2.36–4.95))	133.13 (4.76 (3.54–7.48))	212.82 (7.19 (5.33–11.36))
**Set 5**								
Session 1	109.79 ± 22.85	5.29 ± 2.43	0.61 ± 0.02	1.22 ± 0.11	1965.48 ± 348.43	1180.67 ± 216.30	2806.95 ± 502.57	2909.64 ± 620.73
Session 2	110.07 ± 22.79	5.46 ± 2.15	0.62 ± 0.02	1.27 ± 0.15	1970.82 ± 353.16	1217.07 ± 206.77	2799.91 ± 452.93	3103.23 ± 696.91
TE (CV% (90%CI))	4.77 (4.34 (3.23–6.80))	2.37 (44.16 (31.45–76.32))	0.02 (2.70 (2.01–4.21))	0.05 (4.30 (3.20–6.74))	55.66 (2.83 (2.11–4.42))	52.69 (4.40 (3.27–6.90))	190.37 (6.79 (5.04–10.72))	230.85 (7.68 (5.69–12.16))

Mean ±: Standard Deviation. TE: Typical Error: CV%: Coefficient of Variation. 90% CI: 90% Confidence Interval.

**Table 3:** Kinetic, kinematic, external load, and repetition characteristics from the 30% VLT condition.

	External Load (kg)	Repetitions	Concentric Mean Velocity (m·s^−1^)	Concentric Peak Velocity (m·s^−1^)	Concentric Mean Force (N)	Concentric Mean Power (W)	Concentric Peak Force (N)	Concentric Peak Power (W)
**Set 1**								
Session 1	111.74 ± 24.75	11.24 ± 5.20	0.59 ± 0.02	1.18 ± 0.11	1986.90 ± 354.89	1165.29 ± 227.45	2827.86 ± 535.41	2836.92 ± 595.96
Session 2	110.98 ± 22.25	10.93 ± 6.86	0.61 ± 0.02	1.18 ± 0.12	1974.50 ± 335.19	1183.09 ± 193.35	2781.66 ± 454.45	2760.64 ± 624.73
TE (CV% (90%CI))	3.19 (2.87 (2.14–4.48))	3.23 (29.13 (21.07–48.65))	0.02(3.19 (2.38–4.99))	0.04 (3.27 (2.43–5.11))	29.56 (1.49 (1.11–2.32))	39.53(3.37 (2.51–5.27))	77.19 (2.75 (2.05–4.30))	110.34 (3.94 (2.93–6.18))
**Set 2**								
Session 1	109.09 ± 26.74	8.38 ± 5.40	0.59 ± 0.03	1.17 ± 0.13	1962.47 ± 370.94	1148.61 ± 216.66	2788.33 ± 552.33	2772.43 ± 614.45
Session 2	110.98 ± 22.25	9.33 ± 3.68	0.58 ± 0.03	1.16 ± 0.12	1974.09 ± 335.58	1137.94 ± 210.77	2770.68 ± 451.91	2719.52 ± 581.04
TE (CV% (90%CI))	5.67 (5.16 (3.83–8.11))	2.33 (26.40 (19.15–43.81))	0.02 (3.42 (2.54–5.35))	0.04 (3.59 (2.67–5.62))	49.11(2.49 (1.86–3.90))	27.70 (2.42 (1.81–3.78))	108.20 (3.89 (2.90–6.10))	143.04 (5.21 (3.87–8.19))
**Set 3**								
Session 1	108.79 ± 26.08	7.45 ± 2.09	0.59 ± 0.03	1.20 ± 0.13	1956.44 ± 359.24	1141.54 ± 219.77	2777.24 ± 503.37	2844.29 ± 629.21
Session 2	110.98 ± 22.25	7.66 ± 2.35	0.59 ± 0.02	1.18 ± 0.13	1975.34 ± 338.22	1144.41 ± 21978	2767.18 ± 445.31	2801.82 ± 658.63
TE (CV% (90%CI))	5.71 (5.19 (3.86–8.17))	1.55 (20.46 (14.93–33.46))	0.02 (3.46 (2.57–5.41))	0.06 (4.82 (3.59–7.58))	54.32 (2.76 (2.06–4.32))	29.03 (2.54 (1.89–3.97))	109.15 (3.94 (2.93–6.17))	225.96 (8.00 (5.93–12.68))
**Set 4**								
Session 1	108.16 ± 25.92	8.17 ± 2.38	0.58 ± 0.02	1.18 ± 0.14	1953.63 ± 360.21	1125.64 ± 203.55	2769.06 ± 497.12	2770.82 ± 612.09
Session 2	109.79 ± 21.90	7.23 ± 2.00	0.57 ± 0.03	1.16 ± 0.14	1961.88 ± 329.88	1115.48 ± 187.77	2747.68 ± 439.24	2727.56 ± 619.25
TE (CV% (90%CI))	4.24 (3.89 (2.90–6.10))	1.45(18.92 (13.84–30.83))	0.03(4.69 (3.49–7.37))	0.05 (4.67 (3.47–7.34))	38.54 (1.97 (1.47–3.07))	51.72(4.62 (3.43–7.25))	3.40 (3.20 (2.39–5.01))	168.71 (6.1 (4.56–9.69))
**Set 5**								
Session 1	109.37 ± 23.22	6.63 ± 3.79	0.58 ± 0.04	1.21 ± 0.14	1952.36 ± 359.87	1131.80 ± 213.99	2777.56 ± 503.08	2858.42 ± 607.55
Session 2	108.45 ± 22.43	6.04 ± 1.44	0.58 ± 0.03	1.18 ± 0.13	1950.10 ± 328.88	1127.17 ± 174.59	2753.63 ± 395.70	2766.67 ± 588.53
TE (CV% (90%CI))	2.59 (2.38 (1.78–3.72))	1.74 (27.55 (19.95–45.83))	0.04 (7.38 (5.47–11.68))	0.07 (6.10 (4.53–9.2))	39.79 (2.04(1.52–3.18))	84.42 (7.47 (5.54–11.83))	118.93 (4.30 (3.20–6.75))	215.17 (7.65 (5.67–12.11))

Mean ±: Standard Deviation. TE: Typical Error: CV%: Coefficient of Variation. 90% CI: 90% Confidence Interval.
